# ﻿Bat diversity (Mammalia, Chiroptera) in and around Gomantong and Madai caves, Sabah: insights from field surveys and published records from other Malaysian Bornean caves

**DOI:** 10.3897/zookeys.1249.135209

**Published:** 2025-08-12

**Authors:** Nur Ain Awatif Mohd-Kanapiah, Yen Chi Lok, Nor Azila Sendeng, Muhammad Ali Zulhazim, Mohd Farhan Mohd Johar, Melvin Amandus, Adrian Rawlennes, Noor Haliza Hasan

**Affiliations:** 1 Institute for Tropical Biology and Conservation, University Malaysia Sabah, Sabah, Malaysia University Malaysia Sabah Sabah Malaysia; 2 Faculty of Natural Science and Sustainability, University College Sabah Foundation, Sabah, Malaysia University College Sabah Foundation Sabah Malaysia; 3 Sabah Wildlife Department, Sabah, Malaysia Sabah Wildlife Department, Sabah Malaysia; 4 Sabah Forestry Department, Sabah, Malaysia Sabah Forestry Department Sabah Malaysia

**Keywords:** Biodiversity, Borneo, cave-dwelling bats, Chiroptera, ecology, endemic, Gomantong caves, Madai cave

## Abstract

Gomantong and Madai caves are two of the largest limestone formations with intricate cave systems located in the Lower Kinabatangan and Kunak areas, respectively, in Sabah, Malaysia, Borneo. Despite their ecological and economic significance, limited published information exists on the bat species inhabiting these caves. This study aims to analyze bat diversity at both caves and compare their species richness and diversity to other cave inventories in Sabah and Sarawak. Two bat surveys were conducted around Gomantong caves with a combined trapping effort of 56 trap nights deployed in the surrounding forest. A bat survey at Madai cave utilized 16 trap nights deployed within the caves. A total of 974 and 264 bats were captured from Gomantong and Madai caves, representing 14 and 8 bat species, respectively. A compilation of bat species records from previous studies shows that Gomantong and Madai caves host at least 26 and 30 bat species, respectively. Comparisons with other caves across Malaysian Borneo reveal that Mulu cave and Wind cave in Sarawak, each with 29 species, rank just below Madai cave in bat diversity. Gomantong and Madai caves serve as critical roosting habitats for diverse bat species, including Borneo-endemic and rare species (e.g., *Myotisgomantongensis*, *M.borneoensis*), highlighting their ecological importance and the urgent need for conservation and sustainable management to mitigate anthropogenic threats.

This survey provides an updated checklist of bats in the Gomantong caves and the Madai cave from Sabah, Malaysia. This study also compiles the bat species diversity of other selected caves across Malaysian Borneo.

## ﻿Introduction

Bats (Order Chiroptera) play a vital role in our ecosystems by maintaining the ecology through their role as seed dispersers, pollinators (Baqi et al. 2022), and insect population regulators (Boon and Corlet 1989; Mohd-Azlan et al. 2000; Kasso and Balakrishnan 2013). Chiroptera is the second most speciose order of mammals, after Rodentia, with over 1,400 species described (Senawi et al. 2020; Simmons and Cirranello 2022). Approximately 378 species are known from Southeast Asia (Kuo et al. 2017; Tu et al. 2017; Soisook et al. 2017). Approximately 32% of the bat species can be found in Malaysia, with 99 species reported in Borneo (Sabah and Sarawak, Payne and Francis 1985; Phillipps and Phillipps 2018) and 143 species recorded in Peninsular Malaysia (Senawi and Norhayati 2021). At least ten of the total bat species in Malaysia were classified as Vulnerable by the International Union for the Conservation of Nature (IUCN) in 2022, with one bat species, *Hipposideroscoxi* Shelford, 1901, listed as Endangered in the IUCN Red List since 2016. The populations of some bat species is declining mainly due to habitat loss and hunting (Mickleburgh et al. 2002; Frick et al. 2019), although other factors such as bat disease, wind energy development, and climate changes are also affecting populations in many parts of the world (Welbergen et al. 2008; O’Shea et al. 2016; Frick et al. 2019).

Limestone caves serve as an important habitat for bats (Mohd-Ridwan et al. 2010), with bat guano providing the primary energy source for the cave ecosystem (Chapman 1985; MacKinnon et al. 1996; Hall et al. 2002; Mohd-Ridwan et al. 2010). In temperate countries, caves offer optimal physiological conditions for bats to undergo torpor, hibernate, and conserve energy during the day. In Southeast Asia, caves have been suggested as a critical population reservoir for cave-roosting bats in a fragmented landscape (Struebig et al. 2009). This is particularly true for Borneo’s cave-dwelling bats, which are protected species in the region (Meredith and Wooldridge 1992). All Chiroptera are protected species under Schedule 2 of the Sarawak Wildlife Ordinance 1998, with *Cheiromelestorquatus* Horsfield, 1824 listed as a totally protected species under Schedule 1 of the ordinance. Meanwhile, three species (*Hipposiderosdyacorum* Thomas, 1902, *Pipistrelluscuprosus* Hill & Francis, 1984, and *Murinarozendaali* Hill & Francis, 1984) are protected species with limited hunting and collection under license, Schedule 2 of the Sabah Wildlife Conservation Enactment 1997, while two species (*Pteropusvampyrus* Linnaeus, 1758 and *P.hypomelanus* Temminck, 1853) are under Schedule 3 – protected species of animals for which a hunting license is required.

**Figure 1.**
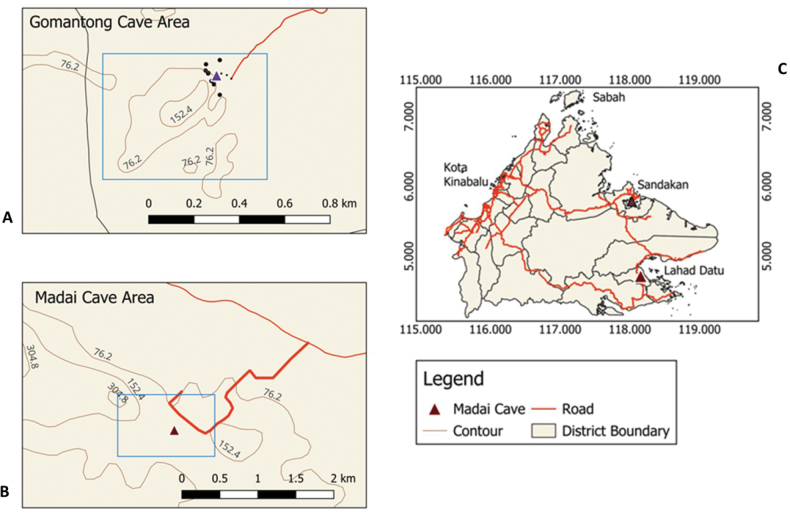
Location of Gomantong Caves within the Gomantong Forest Reserve, Kinabatangan, Sandakan District (A) and Madai Cave within the Madai Baturong Forest Reserve, Kunak, Lahad Datu District (B), with both caves indicated on the Sabah map (C). Harp traps were deployed at the entrance of the Semud Hitam, Gomantong Caves, while mist nets were set up along the boardwalk and forest areas surrounding the cave. At Madai Cave, only harp traps were set up within the cave itself.

Tanalgo et al. (2022) highlighted the importance of caves in hosting endemics, which are a critical ecosystem for their keystone species, bats. Therefore, bats are deemed potential conservation surrogates as umbrella species for taxonomic and spatial conservation of the karst and cave ecosystem. DarkCideS 1.0 is a global database that compiles distribution data and ecological traits of each cave-dwelling species to enable continuous data-sharing to advance bat research in monitoring and conservation prioritisation (Tanalgo et al. 2021). Worldwide, the highest number of cave-dwelling bat species is recorded in China (78 species) and Brazil (71 species) (Tanalgo et al. 2022) (Fig. 1A). Malaysia was shown to have recorded 20 species, specifically from Wind cave and Fairy cave in Bau, Sarawak (Mohd-Ridwan et al. 2011; Rosli et al. 2018; Rajasegaran et al. 2018) and Kota Gelanggi, Jerantut, Pahang (Nurul-Ain et al. 2017) (Fig. 1B).

Increasing numbers of studies have reported bat species checklists from caves throughout Borneo, such as Batu Puteh cave, Madai cave, Niah cave, Wind cave, Fairy cave, Mulu cave, and Mount Silabur cave (Kobayashi et al. 1980; Mohd-Ridwan et al. 2010, 2011; Azhar et al. 2013; Shazali et al. 2017; Rosli et al. 2018; Rajasegaran et al. 2018; [Bibr B81][Bibr B58]). The maintenance of caves is vital for bat conservation, especially in cave-dwelling areas, because bats vary in their selection of roost sites based on the cave zone. For instance, the frugivorous bats in Fairy cave, Bau, Sarawak typically prefer the bright zone, while the insectivorous bats favor the twilight or dark zones (Rajasegaran et al. 2018). Observations of four bat species (*Hipposiderosdiadema* Geoffroy, 1813, *H.larvatus* Horsfield, 1823, *Emballonuramonticola* Temminck, 1838, and *Penthetorlucasii* Dobson, 1880) demonstrated statistically significant differences in roost site selection based on factors such as temperature, humidity, light intensity, airflow, and distance from the entrance of the cave (Rajasegaran et al. 2018).

Gomantong caves are the largest limestone caves in Sabah, located in the 3,297 hectare Gomantong Forest Reserve, Class IV (Amenity Forest), Kinabatangan, Sabah, North Borneo (Sabah Wildlife Department 2021). It is a forest reserve that supports ecotourism for both local and foreign visitors. Since 2012, it has welcomed between 13,000 and 15,000 local and foreign visitors annually, drawn to the harvesting of swiftlet birds’ nests (Ismail 1999; Hobbs 2004; Lundberg and McFarlane 2012), the remarkable bat swarm, and cave guano (Price 2007; Kingston 2010).

Madai cave, on the other hand, is an essential feature of the limestone hills range located within the Madai Baturong Forest Reserve, Class VI (Virgin Forest) in Kunak, Lahad Datu district. This type of forest reserve is strictly designated for forestry research, including ecological baseline research, biodiversity, and genetic conservation, with no timber harvesting permitted in the reserve. This cave is listed in Part I, Schedule 4 of the Wildlife Conservation Enactment 1997, which documents the Ida’an community inheritance rights to harvest swiftlet nests (Sabah Wildlife Department, 2021).

Bat species richness correlates positively with the cave size and is negatively affected by human disturbance (Luo et al. 2013). However, the impact of anthropogenic activities on the Gomantong caves bat population is unclear. Previous bat diversity studies in Gomantong have noted a total of 25 species (Kobayashi et al. 1980; Hill and Francis 1984; Payne and Francis 1985). Of these, four species are classified as Near Threatened according to the IUCN Redlist: *Rhinolophustrifoliatus* Temminck, 1834, *R.sedulus* K. Andersen, 1905, *Kerivoulapellucida* Waterhouse, 1845, and *K.minuta* Miller, 1898, although some of these may roost outside the caves. Additionally, one species has a Vulnerable status: *Hipposiderosridleyi* Robinson & Kloss, 1911. In contrast, a total of 30 species have been reported from the area around Madai cave (Kobayashi et al. 1980; Hill and Francis 1984; Payne and Francis 1985; Mahyudin et al. 2022, including two Vulnerable species: *Rousettusspinalatus* Bergmans & Hill, 1980, and *Hipposiderosridleyi* (Abdullah et al. 2007). Although both caves have included the Vulnerable species *Miniopterusschreibersii* Kühl, 1817, in their species list, recent updates have reidentified the species in Southeast Asia as *Miniopterusblepotis* Temminck, 1840, and its IUCN conservation status has not been assessed (Wilson and Mittermeier 2019).

Therefore, this study aims (i) to provide a compiled checklist of bat species recorded from this study and previous studies for both Gomantong and Madai caves in Sabah, and (ii) to compare the bat species diversity from both locations with another six caves studied in Sabah and Sarawak. These are: one cave in Sabah, Batu Puteh (Supu) cave, Kinabatangan, Sabah (Kobayashi et al. 1980); and six caves from Sarawak: Niah cave, Miri (Mohd Ridwan et al. 2010), Wind cave, Bau (Mohd-Ridwan et al. 2010, 2011; Shazali et al. 2017; Rosli et al. 2018), Fairy cave, Bau (Rajasegaran et al. 2018), Jambusan cave, Bau (Pathe et al. 2005), Mulu cave, Miri (Azhar et al. 2013; Shazali et al. 2016), and Mount Silabur cave, Serian (Jinggong et al. 2022). These data are crucial for monitoring the diversity of bat species in both Gomantong caves and Madai cave, and they will serve as the baseline data for conservation purposes of the forest reserves.

## ﻿Materials and methods

### ﻿Study area

Gomantong caves (5°31'30"N, 118°04'15"E) is located within the 3,297-hectare Gomantong Forest Reserve in Sukau Kinabatangan, Sabah. This complex cave system, managed by the Sabah Wildlife Department, comprises several distinct caves, including the well-known Semud Hitam (Black cave) and Semud Putih (White cave), which are located near each other but are not physically interconnected. The system also includes seven smaller caves, one of which is the type locality for the endemic *Myotisgomantongensis* Francis & Hill, 1998 (Fig. 1). Meanwhile, Madai cave (4°43'23.45"N, 118°9'13.16"E), is situated in southeastern Sabah, within the 3,436.5 hectares of Madai Baturong Forest Reserve, Kunak, Sabah, managed by the Sabah Forestry Department. Both Gomantong and Madai caves are significant sites for swiftlet nest harvesting.

### ﻿Sampling methods

Sampling was conducted during two different sessions around the Gomantong caves (a total of seven nights): 22–25 January 2018 (three nights) and 15–20 March 2021 (four nights). A total of 18 sampling hours were conducted during the first period (22–25 January 2018) and 24 sampling hours during the second period (15–20 March 2021), with ~ 6 h of sampling per day (from 1700 hr to 2300 hr). The sampling was conducted outside the caves, along the forest trail near the streams, potentially at their flyways or roosting sites, and along the boardwalk near the caves. No trapping was done within the cave chambers due to the high ceiling (~40–60 m) of the accessible Simud Hitam. Bats were trapped using six mist nets and two four-bank harp traps (Francis 1989) on all nights (total of 56 net/trap nights). The mist nets were made of nylon, erected using extendable aluminum poles and ropes, with a dimension of 2.5 m height and 9–10 m length. The harp traps were made of aluminum bars with four-bank vertical nylon lines and an attached canvas to trap the bats, which were set up about 1 m above the ground level. However, the 2018 trip had a shorter sampling period due to heavy rain on night four.

Sampling at Madai cave was conducted between 13–17 March 2023 (four nights) from 1600 hr to 2200 hr, totaling six hours per night. Due to the steep structure of the cave surroundings, sampling was conducted using one four-bank harp trap deployed within 3 m of the cave openings, while an additional four-bank harp trap and two mist nets were set up within the cave chamber. The nets were checked every 15–30 minutes until 2200 hr, depending on the capture rate. Only harp traps were left open until the next morning for a final check at 0530 hr. Traps that caught bats were set in the same place for at least two nights, while traps with no captures were moved to a new location. All captured bats were extracted from the nets and traps and temporarily held individually in respective cloth bags for further identification and processing.

### ﻿Sample identification and processing

Captured bats were identified according to Payne and Francis (1985), Kingston et al. (2006), Francis (2008), and Phillipps and Phillipps (2018) only based on external morphology. Identification of bats was done to the species level. The standard morphological measurements, i.e., forearm length (**FA**), ear length (**E**), tibia length (**TB**), hind foot length (**HF**), and tail ventral length (**TVL**), were measured using Mitutoyo digital calipers. The weight was measured using a Pesola spring balance (Hall et al. 2004; Khan et al. 2019).

Some of the bats were photographed for identification purposes and future reference. The gender of each captured individual was identified by the presence of a prominent penis for the males and nipples on both sides for the females. The maturity (juvenile or adult) of the bats was recorded according to Kunz and Kurta (1988) by observing their epiphyseal-diaphyseal fusion on the third, fourth, and fifth metacarpals. Conservation status assessments for the species studied were based on the IUCN Red List of Threatened Species (accessed on 2024).

A maximum of three individuals per species of bat specimens were euthanized using isopropanol following the approval of the Animal Ethics Committee, UMS (AEC-0005/2020). The liver and pectoral muscle were extracted, minced, and preserved in the lysis buffer for further molecular studies (Longmire et al. 1997; Khan et al. 2010). Selected bats were preserved in 70% ethanol as voucher specimens and deposited in the BORNEENSIS Wet Collection of the Institute for Tropical Biology and Conservation, University Malaysia Sabah, while the remaining captured individuals were released after all measurements and photographs were taken.

## ﻿Results

### ﻿Bat species diversity and distribution in Gomantong and Madai caves, Sabah

This study recorded a total of 12 species around Gomantong caves: four species of Pteropodidae, three species of Rhinolophidae, two species of Hipposideridae, one species each for Vespertilionidae, Molossidae, and Miniopteridae (Table 1, Fig. 3). The most abundant species was *Hipposideroscervinus* (*n* = 596), followed by *Rhinolophuscreaghi* Thomas, 1896 (*n* = 317). Singletons recorded include *Eonycterisspelaea* Dobson, 1871, and *Rhinolophusaffinis* Horsfield, 1823. Of the species recorded around Gomantong caves, 11 of 12 are listed as Least Concern on the IUCN Red List, while *Kerivoulaminuta* is listed as Near Threatened.

**Table 1.** List of species recorded and mean measurement (mean, standard deviation (SD), minimum, and maximum ranges) of selected chiropterans at the Gomantong caves of the Gomantong Forest Reserve for both sampling trips (2018 and 2021) and Madai cave of the Madai Baturong Forest Reserve. Note: (^a^ – Gomantong; ^b^ – Madai). No measurement available for *Hipposiderosdiadema* for Madai cave as the presence of this species is through direct observation in the caves.

Family	Species name	Common name	Total	Relative abundance (%)	Forearm (mm)	Ear (mm)	Tibia(mm)	Hindfoot (mm)	Tail ventral length (mm)	Weight (g)
Pteropodidae	*Balionycterismaculata*	Spotted-winged Fruit Bat	4^a^	0.41	43.10 ± 1.28 (41.84–44.79)	12.10 ± 2.33 (8.73–13.75)	15.20 ± 1.43 (13.70–17.19)	8.00 ± 2.05 (4.93–9.24)	na	14.25 ± 0.95 (13.00–15.00)
	*Cynopterusbrachyotis*	Lesser Short-nosed Fruit Bat	3^a^	0.30	59.57 ± 0.94 (58.90–60.24)	13.11 ± 1.45 (12.08–14.14)	21.30 ± 1.11 (20.54–22.11)	7.9 ± 0.68 (7.42–8.39)	na	37.5 ± 2.12 (36.00–39.00)
	*Cynopterusminutus*	Minute Short-nosed Fruit Bat	2^a^	0.20	54.95 ± 1.48 (53.90–56.00)	10.80 ± 0.85 (10.20–11.41)	20.11 ± 0.80 (10.20–11.40)	10.60 ± 2.89 (8.62–12.72)	4.06 ± 1.47 (3.60–5.69)	21.50 ± 0.70 (21.00–22.00)
	*Eonycterisspelaea*	Lesser Dawn Bat	1^a^	0.10	55.30	15.00	20.70	12.20	na	28.00
Hipposideridae	*Hipposideroscervinus*	Fawn-colored Leaf-nosed Bat	596^a^	61.19	46.18 ± 1.16 (45.28–47.79)	10.06 ± 0.91 (9.35–11.09)	15.80 ± 1.61 (14.40–15.50)	6.33 ± 0.61 (5.68–6.89)	21.18 ± 1.48 (20.24–22.89)	8.80 ± 1.93 (7.00–11.5)
			1^b^	2.27	43.83	10.57	14.92	6.58	27.26	
	*Hipposiderosdiadema*	Diadem Leaf-nosed Bat	20^a^	2.05	81.88 ± 2.17 (78.11–84.35)	24.86 ± 6.11 (16.60–30.95)	34.49 ± 1.66 (32.35–37.36)	12.31 ± 1.23 (10.77–14.27)	38.37 ± 6.92 (28.38–49.05)	38.16 ± 3.12 (32.00–40.00)
			30^b^		–	–	–	–	–	–
	*Hipposiderosdyacorum*	Dayak Leaf-nosed Bat	1^b^	2.27	39.88	13.22	16.14	6.22	21.05	Na
Rhinolophidae	*Rhinolophuscreaghi*	Creagh’s Horseshoe Bat	317^a^	32.55	49.40 ± 0.93 (47.86–50.69)	21.08 ± 4.33 (14.60–26.78)	23.16 ± 3.15 (14.72–27.18)	10.96 ± 0.82 (8.08–10.49)	17.00 ± 2.18 (13.38–21.20)	9.79 ± 0.83 (8.00–11.00)
			24^b^	54.55	49.52 ± 1.06 (47.50–51.11)	15.20 ± 1.74 (13.75–17.42)	23.08 ± 1.95 (21.11–25.55)	8.86 ± 0.92 (7.61–9.82)	16.51 ± 2.20 (13.54–18.68)	
	*Rhinolophusborneensis*	Bornean Horseshoe Bat	2^a^	0.20	42.89 ± 0.09 (42.82–42.96)	16.40 ± 3.70 (13.80–19.13)	20.00 ± 3.60 (17.40–22.59)	7.10 ± 0.30 (6.82–7.38)	18.80 ± 4.30 (15.70–21.80)	7.75 ± 0.35 (7.50–8.00)
	*Rhinolophusaffinis*	Intermediate Horseshoe Bat	1^a^	0.10	49.45	24.60	19.97	8.42	19.78	9.00
	*Rhinolophusphilippinensis*	Large-eared Horseshoe Bat	8^b^	18.18	49.17 ± 2.11 (46.30–52.33)	21.65 ± 2.10 (17.90–24.23)	21.11 ± 0.90 (19.36–22.09)	7.57 ± 0.72 (6.87–8.78)	26.79 ± 3.39 (21.07–30.67)	na
Vespertilionidae	*Kerivoulapapillosa*	Papillose Woolly Bat	2^a^	0.20	45.23 ± 1.75 (43.99–46.47)	45.23 ± 1.75 (43.99–46.47)	19.9 ± 2.31 (18.26–21.54)	8.05 ± 0.12 (7.96–8.14)	43.07 ± 3.97 (40.26–45.88)	8.50 ± 2.12 (7.00–10.00)
Mollosidae	*Mopsplicatus*	Wrinkle-lipped Free-tailed Bat	3^a^	0.20	42.25 ± 0.15 (42.10–42.43)	12.48 ± 2.44 (10.19–15.55)	12.69 ± 0.68 (11.82–13.30)	7.79 ± 0.76 (6.85–8.52)	27.39 ± 3.12 (25.02–31.56)	13.00 ± 0.89 (12.00–14.00)
Miniopteridae	*Miniopterusaustralis*	Little Long-fingered Bat	11^a^	1.95	37.05 ± 0.71 (36.35–38.65)	8.82 ± 3.47 (5.43–17.10)	14.71 ± 0.79 (13.50–16.26)	6.78 ± 0.94 (5.32–8.57)	30.01 ± 5.68 (19.40–37.79)	6.36 ± 0.64 (6.00–7.50)
			4^b^	9.09	36.27 ± 1.29 (34.15–37.31)	7.61 ± 0.66 (6.71–8.39)	14.55 ± 0.78 (13.96–15.85)	5.76 ± 0.41 (5.17–6.18)	37.00 ± 4.72 (32.81–43.37)	na
	*Miniopterusmagnater*	Western Long-Fingered Bat	2^b^	4.55	48.31 ± 0.58 (47.90–48.72)	10.35 ± 0.33 (10.12–10.58)	19.67 ± 1.07 (18.91–20.43)	6.94 ± 0.19 (6.80–7.07)	56.83 ± 0.08 (56.77–56.88)	na

When combining the bat species list from this study with previous findings, there are currently 26 known species for Gomantong caves. This includes eight species of Vespertilionidae (30.8%), six species of Pteropodidae (23.1%), five species of Hipposideridae (19.2%), four species of Rhinolophidae (15.4%), two species of Miniopteridae (7.7%), and one species of Molossidae (3.9%) (Table 2).

**Table 2.** Compiled checklist of bat species recorded from the Gomantong caves, Kinabatangan, and Madai cave, Kunak, Sabah. Only individual numbers recorded from this study are indicated in the table. Meanwhile, species recorded from previous studies are only indicated by their presence. Note: * indicates new distributional record based on this study (*n* = 1).

Species	Number of Individuals/Presence (+) Gomantong caves				Number of Individuals/Presence (+) Madai caves				
	This study	Kobayashi et al. (1980)	Hill and Francis (1984)	Payne and Francis (1985)	This study	Kobayashi et al. (1980)	Hill and Francis (1985)	Payne and Francis (1985)	Mahyudin et al. (2022)
**Family Pteropodidae**									
*Cynopterusbrachyotis*	3	-	-	-	0	-	-	-	+
*Cynopterusminutus*	2	-	-	-	0	-	-	-	-
*Cynopterushorsfieldii*	0	-	-	+	0	+	-	-	-
*Balionycterismaculata*	4	-	-	-	0	-	-	+	-
*Eonycterisspelaea*	1	-	-	+	0	-	-	+	-
*Rousettusspinalatus*	0	-	-	-	0	-	-	-	+
*Rousettusamplexicaudatus*	0	-	-	-	0	-	-	+	-
*Penthetorlucasii*	0	-	-	+	0	-	-	-	-
**Family Rhinolophidae**									
*Rhinolophusphilippinensis*	0	+	-	+	8	+	-	+	+
*Rhinolophuscreaghi*	317	+	-	+	243	+	-	+	-
*Rhinolophusborneensis*	2	-	-	+	0	+	-	+	-
Rhinolophusaffinis*	1	-	-	-	0	-	-	-	-
*Rhinolophusfoetidus* (formerly *luctus*)	0	-	-	-	0	-	-	+	-
**Family Hipposideridae**									
*Hipposiderosdiadema*	20	+	-	-	30	+	-	-	+
*Hipposiderosdyacorum*^^	0	-	-	+	1	-	-	-	+
*Hipposideroskingstonae*^	0	-	-	-	0	-	-	+	-
*Hipposideroscineraceus*^	0	-	-	+	0	-	-	+	-
H.cf.saevus (formerly *ater*)^	0	-	+	+	0	-	+	+	-
*Hipposiderosridleyi*	0	-	-	-	0	-	-	-	+
*Hipposiderosgaleritus*^^	0	-	-	+	0	+	-	+	-
*Hipposideroscervinus*^^	596	-	-	+	1	-	-	+	+
*Hipposiderosbicolor*	0	-	+	+	0	-	-	-	-
**Family Vespertilionidae**									
*Kerivoulapapillosa*	2	-	-	-	0	-	-	-	-
*Kerivoulaminuta*	0	-	+	+	0	-	+	+	-
*Kerivoulahardwickii*^^	0	-	-	+	0	-	-	-	-
*Myotishorsfieldii*	0	-	+	-	4	+	-	+	+
*Myotisgomantongensis*^^^^	0	-	-	-	0	-	-	-	-
*Myotismacrotarsus*	0	-	-	-	0	-	-	+	+
*Myotismuricola*	0	-	-	-	0	-	-	-	+
*Myotisborneoensis* (formerly *montivagus*)	0	-	-	-	0	-	-	+	-
*Murinasuilla*	0	-	+	-	0	-	-	-	-
*Murinapeninsularis* (formerly *cyclotis*)	0	-	+	+	0	-	-	-	-
*Murinarozendaali*	0	-	+	+	0	-	-	-	-
*Phoniscusatrox*	0	-	-	-	0	-	+	+	-
**Family Miniopteridae**									
*Miniopterusaustralis*^^^	23	+	-	-	4	+	-	-	(+)
*Miniopterusblepotis* (formerly *schreibersii*)	0	-	-	-	0	-	-	+	+
*Miniopterusmagnater*	0	-	+	+	2	-	-	+	+
**Family Molossidae**									
*Mopsplicatus*	3	+	+	+	0	-	-	+	+
*Cheiromelestorquatus*	0	-	-	-	0	-	-	-	+
**Family Emballonuridae**									
*Emballunoraalecto*	0	-	-	-	0	-	-	-	+
Total number of individuals	974	NA	NA	NA	293	NA	NA	NA	NA
Total number of species	12	5	19	16	8	8	3	17	16
Total number of Family	6	4	6	6	4	5	1	6	7
Overall number of species recorded	26				30				

Taxonomic notes: ^ Wongwaiyut et al. (2023) found that both *Hipposideroskingstonae* and *H.cineraceus* occur at Madai and suggested that the species formerly called *H.ater* should tentatively be called Hipposideroscf.saevus. ^^ Payne and Francis (1985) do not mention specific locations for *H.cervinus*, *H.galeritus*, *H.dyacorum* or *K.hardwickii* but Charles Francis (pers. comm. 31 March 2025) has confirmed that all 3 *Hipposideros* species were recorded from both Gomantong and Madai caves and *K.hardwickii* was caught at Gomantong during surveys in 1982. ^^^ Mahyudin et al. (2022) reported *M.paululus*, but did not report *M.australis* and did not indicate how they distinguished it from *M.australis*, so it is unclear whether both species actually occur there. ^^^^ Hill and Francis (1984) recorded *Myotisaternugax* from Gomantong, but the specimens on which that record were based were subsequently described by Francis and Hill (1998) as a new species, *Myotisgomantongensis*. They were captured at Pasuog Tegas, a small cave to the south of Semud Puteh.

For Madai cave, this study recorded a total of eight species: three species of Rhinolophidae, two species of Hipposideridae, two species of Miniopteridae, and one species of Vespertilionidae (Table 1, Fig. 3). The most abundant species based on capture records was *R.creaghi* (*n* = 243), followed by *Hipposiderosdiadema* (approximate count through direct observation, *n* = 30). Singletons were recorded for *Hipposiderosdyacorum* and *H.cervinus.* All bat species recorded in this study for Madai cave are of Least Concern.

Compilation with previous findings indicates that Madai cave has a known total of 30 bat species (Table 2). This comprises seven species of Vespertilionidae (23.3%), six species each of Hipposideridae (20.0%) and Pteropodid (20.0%), four species each of Miniopteridae (13.3%) and Rhinolophidae (13.3%), two species of Molossidae (6.7%), and single species of Emballonuridae (3.3%).

### ﻿Species accounts

#### ﻿Family Pteropodidae

***Balionycterismaculata* (Thomas, 1893)**

**Material examined.** Malaysia • Sabah, Gomantong caves; 05°31'52.0"N, 118°04'24.6"E; 22–25 January 2018; N.A.A. Mohd-Kanapiah, N.A. Sendeng and N.H. Hasan; collection ID: BOR MAL10148, BOR MAL10154, BOR MAL10167 (*n* = 3 ♀).

**Diagnosis**. This species can be easily identified from its small size with white/ yellowish pale spots on wing membranes, joints, both ear edges, and the front of the eyes (Payne and Francis 1985; Phillipps and Phillipps 2018).

**Conservation status.** Least Concern (Bates et al. 2021).

**Distribution.***Balionycterismaculata* was previously recorded in Sabah (Kota Kinabalu, Madai cave, and Sepilok; Khan et al. 2008) and Sarawak (Batang Ai National Park, Bau, Bako National Park, Kuching, Matang Wildlife Centre, Mount Gading, Mount Dulit, Mount Penrissen, Mulu cave, Niah cave, Samunsam Wildlife Sanctuary, and Sematan; Khan et al. 2008).

**Notes.***Balionycterismaculata* is a forest-dwelling bat and is frequently encountered at higher elevations as well as in lowland dipterocarp forests and mangrove areas (Bates et al. 2021). A total of 12% variation in the cytochrome *b* gene between the Borneo and Peninsular Malaysia populations has led to the revision of the species name of the latter population as *Balionycterisseimundi*, and the former as *B.maculata* (Khan et al. 2008; Lim et al. 2017). Following this finding, *B.maculata* is known to be endemic to Borneo. Deforestation is the main known threat to this species (Hodgkison and Kunz 2006; Bates et al. 2021). Although widespread throughout its range, this species’ population trend is unknown (Bates et al. 2021). There is no direct study on the ecosystem service provided by *B.maculata* to date. Nevertheless, *B.seimundi* is suggested to be a pollen pollinator for *Sonneratia* sp. and *Ceibapentandra* (Mohammad-Shah et al. 2021).

***Cynopterusbrachyotis* (Muller, 1838)**

**Material examined.** Malaysia • Sabah, Gomantong caves; 05°31'48.8"N, 118°04'21.8"E; 15–20 March 2022; N.A.A. Mohd-Kanapiah, Y.C. Lok, M.A. Zulhazim, and M.F.M. Johar; reproductive condition: pregnant; outcome: released (*n* = 3 ♀).

**Diagnosis.** Members of this genus are easily identified through the dog-like face, orange neck fur, white-rimmed ear, and wing bones (Payne and Francis 1985). This species was separated from other similar *Cynopterus* species by measuring their forearm following Phillipps and Phillipps (2018).

**Conservation status.** Least Concern (Csorba et al. 2019).

**Distribution.***Cynopterusbrachyotis* is one of the most common fruit-eating bats in Southeast Asia (Payne and Francis 1985) and South Asia (India, Bhutan, and Bangladesh) (Ul Hasan MA, Kingston T (2022).).

**Notes.***Cynopterusbrachyotis* is a forest-dwelling bat and occupies a wide variety of habitats, including primary, secondary, and burnt mangrove forests, agricultural land, and urban areas (Sheherazade et al. 2017; Lee et al. 2018). This species was previously considered a complex with *Cynopterusminutus*, but can be differentiated by forearm length (> 60 mm for *C.brachyotis*, < 60 mm for *C.minutus*). *Cynopterusbrachyotis* is thought to be able to persist in more open habitats such as forest edges, palm oil plantations, orchards, and gardens as well as wooded areas (Jayaraj et al. 2005; Csorba et al. 2019). Most individuals of this species were captured in mist nets around the forest reserve of Gomantong caves. Currently, no major threat has been identified for this species, but its population trend is unknown (Csorba et al. 2019).

***Cynopterusminutus* Miller, 1906**

**Material examined.** Malaysia • Sabah, Gomantong caves; 05°31'52.0"N, 118°04'24.6"E; 22–25 January 2018; N.A.A. Mohd-Kanapiah, N.A. Sendeng and N.H. Hasan; collection ID: BOR MAL10144, BOR MAL10166 (1 ♂, 1 ♀) (*n* = 2)

**Diagnosis.** This species resembles *C.brachyotis*, but can be distinguished by its smaller size with forearm length < 60 mm.

**Conservation status.** Least Concern (Ruedas and Suyanto 2019).

**Distribution.** This species has been recorded from Indonesia (Sumatra, Nias Island, Java, and Sulawesi) and throughout Borneo (Brunei, Indonesia, Malaysia) (Ruedas and Suyanto 2019).

**Notes.***Cynopterusminutus* is a forest-dwelling bat. Previously, it was considered a complex with *Cynopterusbrachyotis* and was reported to be widespread in distribution. Although *Cynopterusbrachyotis* and *C.minutus* were recorded at the same location in Gomantong during different sampling periods, their habitat preferences appear to differ. *Cynopterusbrachyotis* was more frequently encountered and is known to tolerate a range of habitats, including open areas, forest edges, and disturbed forests (Jayaraj et al. 2012; Ruedas and Suyanto 2019). In contrast, *C.minutus* was only recorded in 2018 and is typically associated with more intact forest conditions, which is consistent with previous studies reporting its occurrence primarily in primary and old secondary forests. *Cynopterusminutus* and *C.brachyotis* are very similar in their external morphological features, but they can be differentiated by examining their forearm lengths. The forearm length of *C.minutus* ranges between 50–60 mm, while that of *C.brachyotis* is between 60–70 mm (Jayaraj et al. 2005, 2012). No major threats have been identified for *C.minutus*, although deforestation is becoming a concern because it is a species that prefers the interior of forests. It has been suggested that its population is decreasing (Ruedas and Suyanto 2019).

***Eonycterisspelaea* (Dobson, 1871)**

**Material examined.** Malaysia • Sabah, Gomantong caves; 05°31'51.0"N, 118°04'21.3"E; 15–20 March 2022; N.A.A. Mohd-Kanapiah, Y.C. Lok, M.A. Zulhazim, and M.F.M. Johar; reproductive condition: pregnant; outcome: released (*n* = 1 ♀)

**Diagnosis.** Easily distinguished from other species of fruit bat by its moderate size, elongated muzzle, and the lack of a claw on its second digit (Payne and Francis 1985).

**Conservation status.** Least Concern (Waldien et al. 2020)

**Distribution.** This species has been recorded throughout Southeast Asia, southern China, and extends west through both northwestern and southern South Asia (Waldien et al. 2020).

**Notes.***Eonycterisspelaea* is a cave-dwelling bat known to form large colonies comprising hundreds to tens of thousands of individuals, sometimes in joint-species colonies with *Rousettusamplexicaudatus* (Waldien et al. 2020). There is a roost of several hundred individuals on the ceiling of Semud Hitam. Although this fruit bat prefers to roost in caves within forested habitats, it has also been observed in mines (Furey et al. 2011), the basements of high-rise buildings (Lim et al. 2015), and the attics of village huts (Waldien et al. 2020). Known as a strong flyer, a record from Malaysia indicates that it can forage as far as 38 km away from its roost (Start and Marshall 1976. This species plays an important role as a pollinator for coastal mangrove species, durian, and tree beans or petai (Harbit et al. 2008; Sritongchuay et al. 2008; Bumrungsri et al. 2009; Bumrungsri et al. 2013; Acharya et al. 2015; Nor Zalipah et al. 2016; Stewart and Dudash 2016; Thavry et al. 2017; Baqi et al. 2022). Despite being reported from various localities in Borneo (Fukuda et al. 2009; Pounsin et al. 2016), its population trend is decreasing due to known threats such as habitat loss and cave disturbances (Waldien et al. 2020).

#### ﻿Family Hipposideridae

***Hipposideroscervinus* (Gould, 1863)**

**Material examined.** Malaysia • Sabah, Gomantong caves; 15–20 March 2022; N.A.A. Mohd-Kanapiah, Y.C. Lok, M.A. Zulhazim, and M.F.M. Johar; collection ID: BOR MAL10116, BOR MAL10115, BOR MAL10117 (*n* = 3 ♂); 22–25 January 2018; N.A.A. Mohd-Kanapiah, N.A. Sendeng and N.H. Hasan; collection ID: BOR MAL10160 (*n* = 596).

Malaysia • Sabah, Madai cave; 13–17 March 2023; N.A.A. Mohd-Kanapiah, N.H. Hasan, and M.F.M. Johar; collection ID: BOR MAL10708 (*n* = 1 ♂).

**Diagnosis.** Recognizable through its broadly triangular ears, and simple nose-leaf with two lateral leaflets and median nose-leaf narrower than the posterior nose-leaf. There are three common fur color variations throughout Borneo, including brown, orange, and grey, with a record of albino form from Bako National Park, Kuching, Sarawak (Naharuddin et al. 2015). This species can potentially be confused with *Hipposiderosgaleritus* Cantor, 1846, but differs in ear shape, tail length, and the width of the median nose-leaf, and can also be distinguished using the cytochrome *b* gene.

**Conservation status.** Least Concern (Armstrong 2021).

**Distribution.***Hipposideroscervinus* has been recorded from Australia, Papua New Guinea, Indonesia, Malaysia, Brunei Darussalam, and the Philippines. This species is recorded throughout Sabah, particularly Madai cave, Kunak; Tawau Hill, Tawau; Crocker Range Park, Keningau; Danum Valley Conservation Area, Lahad Datu.

**Notes.***Hipposideroscervinus* is known to roost in rock crevices and caves (Armstrong 2021). In Borneo, it has been found roosting in large colonies in caves or rock crevices and is often found in small groups (Payne and Francis 1985). This species can be found in various habitats, such as primary and secondary forests, and open areas (Mohd-Ridwan et al. 2011). Cryptic species are suggested to occur throughout their range, especially in the island groups (Armstrong 2021). Currently, there are no major threats to *H.cervinus*, but the species is affected by cave disturbances and forest degradation. Although its population shows a decreasing trend, the decline is insufficient to warrant listing as at risk (Armstrong 2021).

***Hipposiderosdiadema* Geoffrey, 1813**

**Material examined.** Malaysia • Sabah, Gomantong caves; 05°31'52.0"N, 118°04'24.6"E; 22–25 January 2018; N.A.A. Mohd-Kanapiah, N.A. Sendeng and N.H. Hasan; collection ID: BOR MAL10149, BOR MAL10150, BOR MAL10157 (1 ♂, 2 ♀); 15–20 March 2022; N.A.A. Mohd-Kanapiah, Y.C. Lok, M.A. Zulhazim, and M.F.M. Johar; collection ID: BOR MAL10113, BOR MAL10109, BOR MAL10114 (2 ♂, 1 ♀) (*n* = 20).

**Diagnosis.** Identified as the largest *Hipposideros* in Borneo, having three or four lateral leaflets with visible white patches on the shoulders and sides (Payne and Francis 1985). During the surveys at Gomantong caves, *Hipposiderosdiadema* were captured using mist nets near streams in the surrounding forest reserve. At Madai cave, this species was directly observed inside the cave, where 30 individuals or more were found roosting in the cave chambers.

**Conservation status.** Least Concern (Aguilar and Waldien 2021).

**Distribution.***Hipposiderosdiadema* has been recorded from India and most of Southeast Asia (Myanmar, Laos, Vietnam, Thailand, Malaysia, Indonesia, the Philippines, Timor Leste, Solomon Islands, and Papua New Guinea; Aguilar and Waldien 2021). In Borneo, this species has been recorded from Sarawak (Lambir Hills National Park, Kubah National Park and Bako National Park, Niah cave National Park, and Wind cave National Park; Mohd-Ridwan et al. 2010, 2011; Jayaraj et al. 2011; Shazali et al. 2017; Rajasegaran et al. 2018; Rosli et al. 2018), and Sabah (Gomantong cave and Madai cave, Kunak).

**Notes.***Hipposiderosdiadema* is primarily a cave-dweller but has been found in crevices of boulders, tree hollows, and solitary under palm fronds in both primary and secondary forests (Kingston et al. 2006; Lim et al. 2017). Currently, 15 subspecies of this species are known (Aguilar and Waldien 2021). No major threat has been identified for *H.diadema*, although habitat loss and cave disturbances may contribute to the observed decreasing population trend (Aguilar and Waldien 2021).

***Hipposiderosdyacorum* Thomas, 1902**

**Material examined.** Malaysia • Sabah, Madai cave; 13–17 March 2023; N.A.A. Mohd-Kanapiah, N.H. Hasan, and M.F.M. Johar; collection ID: MAL1077 (*n* = 1 ♂).

**Diagnosis.** This species is recognizable from the absence of lateral leaflets, triangular ears, and a simple dark nose-leaf with a narrow internarial septum (Fig. 3E; Payne and Francis 1985). Its forearm is intermediate in size between the smaller *H.cineraceus* (FA 35 mm) and the larger *H.bicolor* (FA 48 mm), with a similar range to *H.kingstonae* (FA 39.0 mm) (Wongwaiyut et al. 2023). The cytochrome *b* gene of this individual was sequenced to confirm its identity as *H.dyacorum*, referencing Wongwaiyut et al. (2023).

**Conservation status.** Least Concern (Khan et al. 2020)

**Distribution.***Hipposiderosdyacorum* is recorded from Peninsular Malaysia (Pulau Pinang, Perak, Terengganu and Kelantan) (Simmons 2005) and throughout Borneo, where it is known from Sarawak (Miri: Mulu National Park (NP), Niah NP, Lambir Hills NP, and Tama Abu; Kapit: Ulu Baleh; Bintulu: Bukit Kana, Kampung Langgir, Engkilili, Similajau NP; Sri Aman: Batang Ai NP; Kuching: Kubah NP, Bako NP, Tanjung Datu NP, Gunung Gading NP, Gunung Regu, Dered Krian NP, Santubong NP), and Sabah (Sandakan: Imbak Canyon; Lahad Datu: Madai Forest Reserve). Additional distribution from Sabah is from Tenom (Mantailang, Crocker Range Park), Tongod (Maliau Basin Conservation Area), and Lahad Datu (Danum Valley Conservation Area).

**Notes.***Hipposiderosdyacorum* is known to roost in caves and under rocks, and previous records have also indicated it may roost in hollow trees. This species is commonly found in lowland rainforests (Payne and Francis 1985). Deforestation and limestone extraction pose threats to *H.dyacorum*. However, due to insufficient data, it cannot be qualified for listing in any threatened category. The population trend of this species remains unknown since it is typically observed in small numbers.

#### ﻿Family Rhinolophidae

***Rhinolophuscreaghi* Thomas, 1896**

**Material examined.** Malaysia • Sabah, Madai cave; 13–17 March 2023; N.A.A. Mohd-Kanapiah, N.H. Hasan, and M.F.M. Johar; collection ID: BOR MAL10698, BOR MAL10694, BOR MAL10695 (2 ♂, 1 ♀); Gomantong caves; 05°31'52.0"N, 118°04'24.6"E; 15–20 March 2022; N.A.A. Mohd-Kanapiah, Y.C. Lok, M.A. Zulhazim, and M.F.M. Johar; collection ID: BOR MAL10145, BOR MAL10155, BOR MAL10158, BOR MAL10159, BOR MAL10161, BOR MAL10162, BOR MAL 10163, BOR MAL 10164, BOR MAL10110, BOR MAL10108, BOR MAL10111, BOR MAL10120 (9 ♂, 3 ♀) (*n* = 317).

**Diagnosis.***Rhinolophuscreaghi* can be identified through the presence of a tuft of stiff hair on the rear of its nose-leaf connecting process (Payne and Francis 1985).

**Conservation status.** Least Concern (Jayaraj 2020a).

**Distribution.***Rhinolophuscreaghi* in Sabah is known from the Madai Forest, Imbak Canyon (Bansa et al. 2020; Senawi et al. 2020), and Pulau Banggi (Jayaraj 2020a). This species was also discovered in Sarawak’s Niah National Park, Mulu National Park, Gunung Gading National Park, and Lesung National Park (Shazali et al. 2018), as well as on Palawan Island in the Philippines (Esselstyn et al. 2004; Jayaraj 2020a).

**Notes.***Rhinolophuscreaghi* is a common cave species known to roost in large colonies, with numbers reaching up to 100,000 in caves (Payne and Francis 1985). Gomantong and Madai caves have very important large roosts of this species. It is common in the primary lowland forest of Palawan, where it often roosts in caves with colonies ranging from hundreds to thousands of individuals (Payne and Francis 1985; Jayaraj 2020a). In Palawan, this species is prevalent in primary lowland forests up to about 700 meters above sea level (Esselstyn et al. 2004). Cave disturbances have been identified as the main threat to this species, and its population is showing a decreasing trend (Jayaraj 2020a).

***Rhinolophusborneensis* Peters, 1861**

**Material examined.** Malaysia • Sabah, Gomantong caves; 05°31'48.9"N, 118°04'21.9"E; 15–20 March 2022; N.A.A. Mohd-Kanapiah, Y.C. Lok, M.A. Zulhazim, and M.F.M. Johar; reproductive condition: Pregnant; outcome: released (*n* = 1), collection ID: BOR MAL10121 (*n* = 2 ♂).

**Diagnosis.** Distinguished by its relatively small size (FA 40–44) and small, rounded connecting process (Payne and Francis 1985).

**Conservation status.** Least Concern (Jayaraj 2020b).

**Distribution.***Rhinolophusborneensis* is known from Malaysia (Sabah, Sarawak) (Jayaraj 2020b).

**Notes.***Rhinolophusborneensis* is a cave-dweller and typically roosts in caves with colonies of several hundred individuals and inhabits both primary and secondary forests (Payne and Francis 1985; Rosli et al. 2018). Although this species sometimes shows solitary roosting behavior (Rosli et al. 2018) , there are records of it roosting together with *Rhinolophusaffinis* in caves with large colonies, including those in Gomantong cave, Madai cave, and Mount Kinabalu (Payne and Francis 1985). Formerly thought to occur in Indochina, but genetic studies have shown those records belong to a distinct species, *Rhinolophuschaseni* Sanborn, 1939 (Francis 2008). Currently, no major threats to this species have been identified, and its population trend remains unknown (Jayaraj 2020b).

***Rhinolophusaffinis* Horsfield, 1823**

**Material examined.** Malaysia • Sabah, Gomantong caves; 05°31'52.0"N, 118°04'24.6"E; 22–25 January 2018; N.A.A. Mohd-Kanapiah, N.A. Sendeng and N.H. Hasan; collection ID: BOR MAL10152 (*n* = 1 ♂).

**Diagnosis.** Characterized by its relatively long forearm (~50 mm) and rounded connecting process (Payne and Francis 1985). The identification was confirmed by analysis of mtDNA.

**Conservation status.** Least Concern (Furey et al. 2020).

**Distribution.***Rhinolophusaffinis* is a widespread and common species found distributed throughout Asia, ranging from India to Southeast Asia (Payne and Francis 1985; Corbet and Hill 1992; Wilson and Mittermeier 2019). However, there are relatively few records from Sabah, and this is the first confirmed record from Gomantong.

**Notes.***Rhinolophusaffinis* is known as a cave-dwelling bat that inhabits open and cultivated areas within primary and secondary forests. It typically roosts in caves, often forming enormous colonies, but has also been found in buildings, hollow trees, and foliage (Walston et al. 2008). Despite its preference for caves, where it often forms sizable colonies (Ith et al. 2016) , it also forages in the forest understory, from highly disturbed areas to mature lowland rainforests (Francis 2019). Currently, no major threats are known for this species due to its adaptive nature and stable population trend (Furey et al. 2020).

***Rhinolophusphilippinensis* Waterhouse, 1843**

**Material examined.** Malaysia • Sabah, Madai cave; 13–17 March 2023; N.A.A. Mohd-Kanapiah, N.H. Hasan, and M.F.M. Johar; collection ID: BOR MAL10693, BOR MAL10696, BOR MAL10699 (1 ♂, 2 ♀) (*n* = 3).

**Diagnosis.** This species is easily identified by its very large ears and large protruding nose-leaf (Payne and Francis 1985).

**Conservation status.** Least Concern (Armstrong 2021).

**Distribution.***Rhinolophusphilippinensis* has been recorded from the Philippines, Indonesia, the Kai Islands of West Papua, and Australia (Armstrong 2021). In Borneo, this species has been recorded from Sabah (Sandakan: Gomantong cave, Lahad Datu: Madai cave and Tepadung cave; and Nabawan: Sapulut); and Sarawak (Miri: Baram River, Niah National Park; and Bintulu).

**Notes.** This species roosts in caves and forages within the forest interior. Different size forms of this species have been documented across various parts of its range, including in Buton Island, Sulawesi (Kingston and Rossiter 2004); Papua New Guinea (Armstrong 2017); and Cape York Peninsula, Queensland (Churchill 2008; Armstrong 2021). In Australia, two distinct taxa formerly referred to as this species have been identified: *Rhinolophusrobertsi* Tate, 1952 and *R. philippinensis ‘intermediate*’ (Woinarski et al. 2014). Although it is an uncommon species in most of its distribution, often captured in low numbers or as single individuals, there are moderately large colonies present in Sabah (Armstrong 2021). In our survey, *R.philippinensis* were captured in harp traps in the Madai cave. While forest loss and degradation are the primary threats to this species, its population trend remains unknown due to insufficient data.

#### ﻿Family Vespertilionidae

***Kerivoulapapillosa* (Temminck, 1840)**

**Material examined.** Malaysia • Sabah, Gomantong caves; 05°31'52.0"N, 118°04'24.6"E; 22–25 January 2018; N.A.A. Mohd-Kanapiah, N.A. Sendeng and N.H. Hasan; collection ID: BOR MAL10165 (*n* = 1 ♂); 15–20 March 2022; N.A.A. Mohd-Kanapiah, Y.C. Lok, M.A. Zulhazim, and M.F.M. Johar; collection ID: BOR MAL10118 (*n* = 1 ♀).

**Diagnosis.** The genus is easily recognizable by its woolly fur and funnel-shaped ears with long, pointed tragus (Payne and Francis 1985). This is the largest species in the genus.

**Conservation status.** Least Concern (Hutson and Kingston 2021).

**Distribution.***Kerivoulapapillosa* is known from Malaysia (Sabah and Sarawak), Brunei Darussalam, Indonesia (Kalimantan, Sumatera, Java, Sulawesi), Thailand, Laos, Vietnam, and Cambodia (Hutson and Kingston 2021). Notes. *K.papillosa* is a species reliant on forests (forest-dwellers), which confines its distribution to areas with suitable roosts (Struebig et al. 2009). It typically inhabits lowland mixed-deciduous dipterocarp forests and is found in groups of 1–14 individuals (Hutson and Kingston 2021). The species faces threats from logging activities (Joann et al. 2011). Both molecular data (Khan et al. 2010) and morphological evidence (Hasan and Abdullah 2011) suggest the existence of a species complex within *Kerivoulapapillosa*. Currently, its population trend is considered stable (Hutson and Kingston 2021).

***Myotishorsfieldii* (Temminck, 1840)**

**Material examined.** Malaysia • Sabah, Madai cave; 13–17 March 2023; N.A.A. Mohd-Kanapiah, N.H. Hasan, and M.F.M. Johar; collection ID: BOR MAL10702, BOR MAL10705 (*n* = 2 ♂).

**Diagnosis.** This species can be identified as Myotis based on its general shape of the ear and tragus, with moderately large feet with wing membrane attached to the side of foot, as least 1 mm from base of toes (Payne and Francis 1985).

**Conservation status.** Least concern (Phelps et al. 2019).

**Distribution.***M.horsfieldii* is recorded from India, Singapore, Myanmar, Laos, Thailand, Cambodia, Vietnam, China, Hong Kong, the Philippines, Indonesia (Bali, Kalimantan, Jawa, Sulawesi), Brunei, and Malaysia (Phelps et al. 2019). In Borneo, this species has been recorded from Sabah (Kinabatangan: Sukau, Lahad Datu: Madai and Tepadong), and Sarawak (Miri: Niah) Phillipps and Phillipps (2018).

**Notes.** This species is known to roost in the crevices of bell holes in caves located near rivers or large streams (Payne and Francis 1985). This may likely be a species complex, with individuals from Indochina and India potentially representing distinct, closely related species (Phelps et al. 2019). Commonly recognized throughout its range, its population remains stable.

#### ﻿Family Molossidae

***Mopsplicatus* (Buchannan, 1800) (Previously known as *Tadaridaplicata* , *Chaerephonplicata*)**

**Material examined.** Malaysia • Sabah, Gomantong caves; 05°31'43.7"N, 118°04'24.6"E; 15–20 March 2022; N.A.A. Mohd-Kanapiah, Y.C. Lok, M.A. Zulhazim, and M.F.M. Johar; reproductive condition: pregnant; outcome: released (*n* = 2 ♀).

**Diagnosis.** Easily identified by its heavily wrinkled upper lip, and thick tail that protrudes from the interfemoral membrane (Payne and Francis 1985).

**Conservation status.** Least Concern (Csorba et al. 2020).

Distribution. This species has only been recorded in Borneo from Mulu National Park in Sarawak and Gomantong caves in Sabah (Csorba et al. 2020). No major threats have been identified, but it may be affected by deforestation and cave disturbances. It has an unknown population trend (Csorba et al. 2020).

**Notes.***Chaerephonplicatus* is a cave-dwelling bat that forms large colonies, ranging from hundreds to tens of thousands of individuals (Payne and Francis 1985; McFarlane et al. 2015; Csorba et al. 2020). There is a very large colony roosting in the back of Semud Hitam in a chamber known as Agob Kabilau, with possibly up to 1 million individuals. Most of these bats emerge from the caves very high up, and fly high into the sky. This would explain why we caught so few individuals, with one in a mist net at the starting trail entering Gomantong forest reserve, and another at the boardwalk near the office of the Gomantong Forest Reserve.

#### ﻿Family Miniopteridae

***Miniopterusaustralis* Tommes, 1858**

**Material examined.** Malaysia • Sabah, Gomantong caves; 05°31'46.2"N, 118°04'23.2"E; 15–20 March 2022; N.A.A. Mohd-Kanapiah, Y.C. Lok, M.A. Zulhazim, and M.F.M. Johar; collection ID: BOR MAL10112, BOR MAL10107, BOR MAL10119, BOR MAL10146, BOR MAL10147, BOR MAL10153, BOR MAL10156, BOR MAL10697, BOR MAL10703 (6 ♂, 3 ♀) (*n* = 19).

**Diagnosis.** This genus is characterized by its wing shape, where its third phalanx has a very long terminal phalanx and short subterminal phalanx; its ear is short and rounded with a posterior fold and a short, blunt tragus that curves slightly forward (Payne and Francis 1985). Fur is short, dense, and velvety, giving it a sleek and well-groomed appearance, and very dark brown with a yellowish tip (Fig. 3J). Its ear is observably translucent, allowing light to pass through. The species of *Miniopterus* can only reliably be distinguished by measurements.

**Conservation status.** Least Concern (Armstrong 2021).

**Distribution.***Miniopterusaustralis* often roosts in a large colony, although it could also be found to roost individually (Payne and Francis 1985). This species is widely distributed, with a stable population trend, and no major threats have been identified apart from cave disturbances; hence, it is listed as Least Concern (Armstrong 2021). Due to its widespread distribution, a potential species complex is suggested throughout its range, and ongoing taxonomic work is in progress (Armstrong 2021).

**Notes.** This is a very common species in some parts of Simud Hitam. During our survey (15–20 March 2022), we caught and released a total of 16 individuals of pregnant *Miniopterusaustralis*, which were captured in our harp traps near Semud Hitam, Gomantong cave.

***Miniopterusmagnater* Sanborn, 1931**

**Material examined.** Malaysia • Sabah, Madai cave; 13–17 March 2023; N.A.A. Mohd-Kanapiah, N.H. Hasan, and M.F.M. Johar; collection ID: BOR MAL10704 (*n* = 1 ♂).

**Diagnosis.** This species is similar to *M.australis*, but much larger. We found it had short and dense fur, slightly disheveled and untamed, different from those of *M.australis*, with brownish pelage (Fig. 3K). The ear is opaque and solid in appearance, with no light passing through its surface.

**Conservation status.** Least concern (Armstrong 2021).

**Distribution.***M.magnater* is recorded from India, Myanmar, Thailand, Laos, Cambodia, Myanmar, Vietnam, China, Malaysia, and Papua New Guinea (Armstrong 2021). In Borneo, this species is known from Sabah (Kudat: Balambangan cave, Ranau: Kinabalu Park and Poring, Sandakan: Gomantong cave, upper Sungai Kuamut, Lahad Datu: Madai cave) (Payne and Francis 1985).

**Notes.***Miniopterusmagnater* is a cave species and has been observed foraging near street lights in Kinabalu Park (Payne and Francis 1985). It is a common species, but further work is required on its taxonomy to determine if the species in Borneo is the same as the species on mainland SE Asia (Appleton et al. 2004; Tian et al. 2004).

### ﻿Bat diversity and distribution across nine caves from Sabah and Sarawak

**Figure 2.**
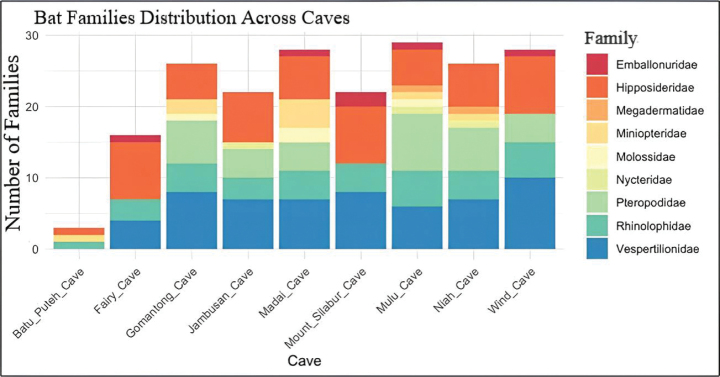
General families presence distribution for each cave based on families showed that all nine caves are dominated by the Hipposideridae, Rhinolophidae and Vespertilionidae.

Results from this survey for each respective cave were compared with results from previous studies from Gomantong caves (Kobayashi et al. 1980; Hill and Francis 1984; Payne and Francis 1985; Francis and Hill 1998) and Madai cave (Kobayashi et al. 1980; Hill and Francis 1984; Payne and Francis 1985; Mahyudin et al. 2022). Additionally, we compared bat diversity from other cave studies in Sabah and Sarawak using presence data from one other cave in Sabah, namely Batu Puteh (Supu) cave, Kinabatangan (5°28'22.292"N, 117°55'20.571"E) (Kobayashi et al. 1980), and another six (6) caves from Sarawak: Camp 5 Mulu cave, Miri (4°07'55.2"N, 114°55'08.4"E) (Azhar et al. 2013), Niah cave, Miri (3°48'54.3522"N, 113°46'29.6004"E) (Mohd-Ridwan et al. 2010), Mount Silabur cave, Serian (00°57.291'N, 110°30.223'E) (Jinggong et al. 2022), Jambusan cave, Bau (1°24'6.5298"N, 110°11'24.3018"E) (Pathe et al. 2005, Fairy cave, Bau (1°22'55.2282"N, 110°7'3.738"E) (Rajasegaran et al. 2018), Wind cave (1°24'51.4002"N, 110°8'15.0498"E) (Mohd-Ridwan et al. 2010, 2011; Shazali et al. 2017; Rosli et al. 2018) (Fig. 2, Table 3).

**Figure 3.**
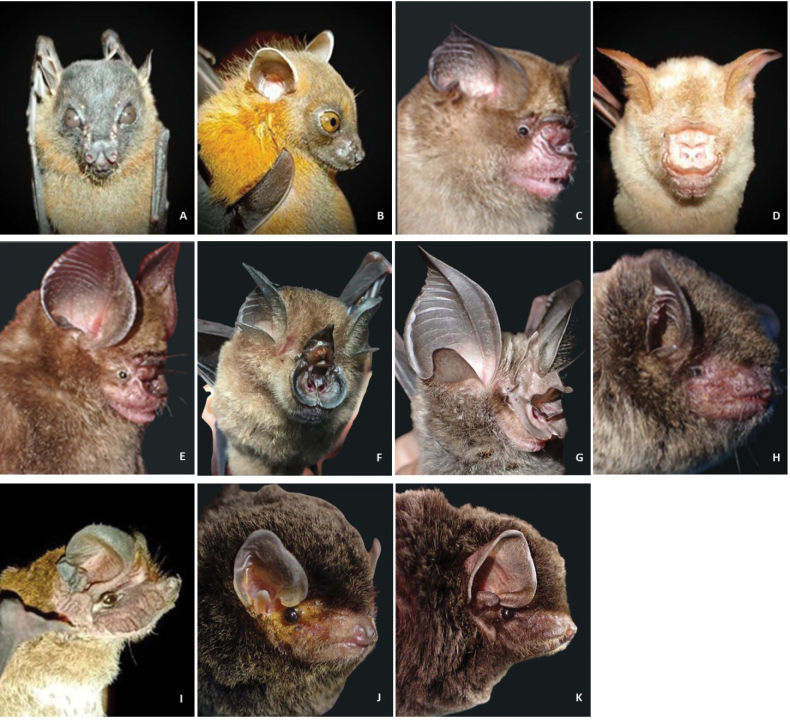
Selected bat profile photos of species recorded at Gomantong Caves and Madai Cave. A. *Balionycterismaculata*; B. *Cynopterusbrachyotis*; C. *Hipposideroscervinus*; D. *Hipposiderosdiadema*; E. *Hipposiderosdyacorum*; F. *Rhinolophuscreaghi*; G. *Rhinolophusphilippinensis*; H. *Myotishorsfieldii*; I. *Chaerephonplicatus*; J. *Miniopterusaustralis*; K. *Miniopterusmagnater.* Photo credits NH Hasan 2023 (C, E–H, J, K); NAAMK 2018, 2021 (A, B, D, I).

**Table 3.** Bat species compilation (*n* = 62) for a total of nine caves across Sabah and Sarawak, Malaysian Borneo. The highest number of species recorded so far is for Madai cave, Sabah, followed by Mulu cave and Wind cave, Sarawak; while the least species number recorded for Batu Puteh cave, Sabah. This may reflect variation in effort as well as species composition. IUCN conservation status is based on data checked in 2024, while each species status as cave (c) (*n* = 22) or forest (f) (*n* = 22) specialist, or known to roost in both forest and caves (*n* = 17) is annotated based on reference (**1**) Payne and Francis (1985), (**2**) the IUCNRedlist.org, and (15) Wilson and Mittermeier (2019). References: Gomantong and Madai caves species list are based on compilation listed on Table 2 above; other caves are from: (**3**) Kobayashi et al. 1980, (**4**) Mahyudin et al. 2022, (**5**) Hill and Francis 1984, (**6**) Mohd-Ridwan et al. 2010, (**7**) Mohd- Ridwan et al. 2011, (**8**) Shazali et al. 2017, (**9**) Rosli et al. 2018, (**10**) Rajasegaran et al. 2018, (**11**) Azhar et al. 2013, (**12**) Jinggong et al. 2022, (**13**) Pathe et al. 2006, (**14**) Wongwaiyut et al. 2023.

Species	Common Name	Presence (+) per cave locality									IUCN Status (2022)	Cave (c)/ Forest (f)
		Sabah			Sarawak							
		Gomantong cave	Madai cave	Batu Puteh cave	Niah cave	Wind cave	Fairy cave	Mulu cave	Mount Silabur Cave	Jambusan cave		
	References	This study (3–5, 14)		(3)	(6)	(6–9)	(10)	(11)	(12)	(13)	(2)	(1, 2, 15)
**Family Pteropodidae**												
*Cynopterusbrachyotis*	Sunda Short-nosed Fruit Bat	+	+		+	+		+		+	LC	f
*Cynopterushorsfieldii*	Horsfield’s Short-nosed Fruit Bat	+	+					+			LC	f
*Cynopterusminutus*	Forest Short-nosed Fruit Bat	+									LC	f
*Balionycterismaculata*	Spotted-winged Fruit Bat	+			+	+		+		+	LC	f
*Eonycterisspelaea*	Lesser Nectar Bat	+			+	+					LC	c
*Rousettusspinalatus*	Bare-backed Rousette		+								VU	c
*Rousettusamplexicaudatus*	Geoffroy’s Rousette		+		+						LC	c
*Dyacopterusspadiceus*	Dayak Fruit Bat				+			+			NT	f
*Penthetorlucasii*	Dusky Short-nosed Fruit Bat	+			+	+		+		+	LC	c
*Megaeropsecaudatus*	Sunda Tailless Fruit Bat							+			LC	f
^*Megaeropsalbicollis* (formerly *wetmorei*)	White-collared Fruit Bat							+			VU	f
*Macroglossusminimus*	Lesser Long-nosed Fruit Bat							+		+	LC	f
**Family Rhinolophidae**												
*Rhinolophusphilippinensis*	Large-eared Horseshoe Bat	+	+		+			+	+		LC	c
*Rhinolophuscreaghi*	Creagh’s Horsehoe Bat	+	+	+	+	+		+			LC	c
*Rhinolophusborneensis*	Bornean Horseshoe Bat	+	+		+	+	+	+	+		LC	c,f
*Rhinolophusaffinis*	Intermediate Horseshoe Bat	+				+		+	+	+	LC	c,f
*Rhinolophustrifoliatus*	Trefoil Horseshoe Bat				+						NT	f
*Rhinolophussedulus*	Lesser Woolly Horseshoe Bat					+	+			+	NT	f
*Rhinolophusfoetidus*	Great Woolly Horseshoe Bat		+			+	+	+	+	+	LC	c,f
**Family Megadermatidae**												
*Megadermaspasma*	Lesser False Vampire Bat				+			+			LC	c,f
**Family Nycteridae**												
*Nycteristragata*	Malayan Slit-faced Bat				+			+		+	NT	c,f
**Family Hipposideridae**												
*Hipposiderosdiadema*	Diadem Leaf-nosed Bat	+	+		+	+	+	+	+	+	LC	c,f
*Hipposiderosdyacorum*	Dayak Leaf-nosed Bat	+	+			+	+	+	+	+	LC	c
*Hipposideroskingstonae*	Kingston’s Leaf-nosed Bat,	+	+		+	+	+		+	+	LC	c,f
Hipposideroscf.cineraceus*	Kingston’s Leaf-nosed Bat,		+								LC	c,f
Hipposideroscf.saevus (formerly *ater*)	Dusky Leaf-nosed Bat		+								LC	c,f
*Hipposiderosridleyi*	Ridley’s Leaf-nosed Bat		+			+					VU	c,f
*Hipposiderosgaleritus*	Cantor’s Leaf-nosed Bat		+	+	+	+	+	+	+	+	LC	c
*Hipposideroscervinus*	Fawn-colored Leaf-nosed Bat	+	+		+	+	+	+	+	+	LC	c
*Hipposideroslarvatus*	Intermediate Leaf-nosed Bat					+	+		+	+	LC	c
*Hipposiderosbicolor*	Bicolored Leaf-nosed Bat	+			+		+	+	+	+	LC	c,f
*Hipposideroscoxi*	Cox’s Leaf-nosed Bat					+	+		+		EN	c
*Coelopsrobinsoni*	Malayan Tailless Leaf-nosed Bat				+						VU	c,f
**Family Vespertilionidae**												
*Kerivoulapapillosa*	Papillose Woolly Bat	+			+			+	+		LC	f
*Kerivoulapellucida*	Clear-winged Woolly Bat				+	+	+	+			NT	f
*Kerivoulaminuta*	Least Woolly Bat	+	+					+			NT	f
*Kerivoulahardwickii*	Hardwicke’s Woolly Bat				+			+	+		LC	f
*Kerivoulaintermedia*	Small Woolly Bat					+	+		+	+	NT	f
*Myotisater*	Peter’s Myotis	+	+		+	+			+	+	LC	c
*Myotishorsfieldii*	Horsfield’s Myotis	+	+		+	+		+	+	+	LC	c
*Myotisgomantongensis*	Gomantong Myotis	+									LC	c
*Myotismacrotarsus*	Pallid Large-footed Myotis		+								LC	c
*Myotismuricola*	Nepalese Whiskered Myotis		+			+	+	+		+	LC	c,f
*Myotisborneoensis*	Bornean Whiskered Myotis		+			+			+		DD	c
*Myotishasseltii*	Lesser Large-footed Myotis					+	+				LC	c,f
*Myotisridleyi*	Ridley’s Myotis									+	NT	c
*Murinasuilla*	Brown Tube-nosed Bat	+							+		LC	f
*Murinapeninsularis* (formerly *cyclotis*)	Peninsular Tube-nosed Bat	+									LC	c,f
*Murinarozendaali*	Gilded Tube-nosed Bat	+			+						VU	f
*Pipistrellustenuis*	Least Pipistrelle									+	LC	f
*Phoniscusatrox*	Groove-toothed Trumpet-eared Bat		+								NT	f
*Tylonycterisrobustula*	Greater Bamboo Bat					+					LC	f
*Tylonycterispachypus*	Lesser Bamboo Bat					+				+	LC	f
*Philetorbrachypterus*	Rohu’s Bat				+						LC	f
*Glischropustylopus*	Common Thick-thumbed Bat					+			+		LC	f
**Family Miniopteridae**												
*Miniopterusaustralis*	Little Long-fingered Bat	+	+	+	+			+			LC	c
^*Miniopterusblepotis* (formerly *schreibersii*)	Javanese Long-fingered Bat		+								VU	c
*Miniopterusmagnater*	Western Long-fingered Bat	+	+								LC	c
**Family Molossidae**												
*Mopsplicatus*	Wrinkle-lipped Free-tailed Bat	+	+					+			LC	c
*Cheiromelestorquatus*	Greater Naked Bat		+								LC	c
**Family Emballonuridae**												
*Emballonuraalecto*	Small Asian Sheath-tailed Bat		+			+		+	+		LC	c,f
*Emballonuramonticola*	Lesser Sheath-tailed Bat						+		+		LC	c,f
Total Species Number Recorded		26	30	3	27	29	16	29	22	24		
Total Family Number		6	7	3	7	5	4	9	4	5		

*Some records of *H.cineraceus* may represent *H.kingstonae* due to unresolved taxonomy. Older records identified as *H.ater* are now considered to belong to H.cf.saevus.

Madai cave has the highest recorded number of bat species (*n* = 30), followed by Mulu cave (*n* = 29) and Wind cave (*n* = 29). In contrast, Batu Puteh cave has the lowest recorded number of species (*n* = 3) (Fig. 2, Table 3). The Hipposideridae, Rhinolophidae, and Vespertilionidae were the three most commonly observed bat families across all nine caves. The most commonly found species across all nine caves were *Hipposideroscervinus*, *H.galeritus*, and *H.diadema*, which were found in eight of the nine caves. *Rhinolophusborneensis*, *Hipposiderosdyacorum*, H.cf.saevus (formerly *H.ater*), and *Myotishorsfieldii* were reported from seven caves.

## ﻿Discussion

### ﻿Cave as the key ecosystem for endemic and rare bat species in Malaysian-Borneo

The area around Madai cave has the highest number of bat species documented to date, followed by Mulu and Wind caves. These caves are predominantly inhabited by species from the families Hipposideridae, Rhinolophidae, and Vespertilionidae, which are among the most speciose families with many species closely associated with caves as roosting sites (Payne and Francis 1985; Phillipps and Phillipps 2018). Notably, each of these caves was sampled for at least 14 nights per locality (Kobayashi et al. 1980; Mohd-Ridwan et al. 2010, 2011). Such prolonged sampling efforts likely enhanced species detectability at these sites (Meyer et al. 2011, 2015), thereby influencing the observed species diversity.

It should be noted that the species listed in Table 3 are not exclusively cave-roosting species, as sampling also included the forests surrounding the cave areas (Hill and Francis 1984; Payne and Francis 1985). Some species are known to roost both in forests and caves, while others recorded at only one cave locality may be roosting in parts of the cave that are poorly sampled (e.g., *Rousettusspinalatus* Bergmans & Hill, 1980, *Miniopterusblepotis* Temminck, 1840, and *Cheiromelestorquatus*, Horsfield, 1824) or forest-dwelling species captured from adjacent forest habitats (e.g., *Cynopterusminutus* Miller, 1906, *Megaeropsecaudatus* Temminck 1837, *Rhinolophustrifoliatus* Temminck 1834, *Pipistrellustenuis* Temminck 1840, *Phoniscusatrox* Miller, 1905, *Tylonycterisrobustula* Thomas, 1915, and *Philetorbrachypterus* Temminck, 1840).

The presence of water bodies near the cave systems may also contribute to species occurrences. For instance, *Myotisridleyi* Thomas, 1898 is frequently reported to forage and roost near water bodies (Azhar 2020). Furthermore, sampling efforts biased toward localities within protected areas may not fully encompass the complete ranges of some species or provide equitable representation across taxa (Fisher-Phelps et al. 2017).

### ﻿Significance of rare and endemic species

Among the documented species, three Borneo endemics (*Hipposideroscoxi*, *Myotisgomantongensis*, *Myotisborneoensis*) (Francis and Hill 1998; MacArthur 2016; Görföl and Csorba 2017; Waldien et al. 2021) highlight the conservation importance of these caves. Limited ecological knowledge about these species, coupled with 23 of the 63 species showing a declining population trend on the IUCN Red List, emphasizes the need for conservation prioritization. Additionally, several species require reassessment of their conservation status due to taxonomic revisions (e.g., *Megaeropsalbicollis*, *Hipposideroskingstonae*, and *Murinapeninsularis*) (Wilson and Mittermeier 2019; Wongwaiyut et al. 2023; Simmons and Cirranello 2024).

### ﻿Trapping bias and methodological considerations

Species detectability is influenced by sampling methods and trap deployment strategies (Meyer et al. 2015). Harp traps captured 89.5% of bats in this study, effectively targeting small insectivorous species, while mist nets were more suitable for frugivorous bats. However, mist nets are limited by “net avoidance” behaviors (Tuttle 1974; Kunz and Kurta 1988), and researchers must be experienced in their use to avoid injuries in captured bats (Breviglieri and Pedro 2010). Traditional trapping methods, such as mist nets and harp traps, while effective, have inherent limitations that influence species detectability.

Insectivorous bats such as *H.diadema*, with larger body sizes, were occasionally caught using mist nets, but smaller species often escaped due to their agility and sharp molars. Nevertheless, there are a few observations where insectivorous bats were recorded in both trapping methods in the current study, namely, *M.australis* and *K.papillosa*.

The integration of acoustic surveys in future studies could complement traditional trapping methods by identifying species underrepresented in live captures (de Aguiar Silva et al. 2022). For example, acoustic methods have detected species typically associated with forests in more open areas (*Kerivoulapapillosa* and *Rhinolophusborneensis*; Yoh et al. 2020). This suggests that advancements in acoustic techniques could improve the detection of cryptic or elusive species. However, no acoustic device was deployed in any of the surveys due to its unavailability at the time, and it will be considered in future works.

The distribution of bats is generally influenced by factors such as forest type or foraging habitat, resource abundance, diet and feeding strategies, and the availability of suitable roosting sites (Tuen et al. 2000; Mohd-Ridwan et al. 2018). Sampling biases in trapping designs, such as focusing on specific forest or cave areas, may skew findings (Meyer et al. 2015). For example, traps placed in open forest understory flyways may capture species like *Hipposideros* spp. and *Rhinolophus* spp., while overlooking species that prefer forest edges, such as *Hypsugovordermanni* (Hasan et al. 2022), or those that forage in or above the forest canopies, such as *Emballonura* spp. or *Mops* spp. (Denzinger and Schnitzler 2013). Similarly, *Myotis* spp. and *Hypsugo* spp., known as edge-space trawling foragers that fly low over water bodies (Denzinger and Schnitzler 2013), may be underrepresented in studies where traps are not positioned above these habitats. Conversely, traps placed at lower cave openings may capture species like *Rhinolophuscreaghi*, while missing those emerging from higher cave openings, such as *Tadarida* spp. (Frick et al. 2012; Denzinger and Schnitzler 2013).

During our sampling, *H.diadema* were observed perching on the ceiling and walls of the first chamber in Madai cave. However, none were captured using the mist nets deployed across the chamber, positioned along one of the potential flight pathways. It is plausible that they favor a higher opening for their emergence flights, or their lowest flight threshold is more than four meters in height. However, this is an assumption made based on a brief observation in the field with no actual record of flight takeoff trajectory or flight pathways.

### ﻿Proposed future studies on bat diversity in Gomantong and Madai caves, Sabah

Given the unique ecological significance of Gomantong and Madai caves, the largest cave systems in Sabah, Malaysia, a comprehensive survey for future studies combining eDNA analysis with acoustic monitoring is necessary to fully understand their bat diversity. Environmental DNA (eDNA) analysis refers to the materials obtained from environmental samples rather than directly from the organisms themselves. This includes samples of DNA that can be found in soil, water, air, and other substrates where organisms interact with their environment. The study by Garret et al. (2023) found that airborne eDNA sampling accurately characterized a diverse community of bats, recovering over 91% of the species present in the sampled area. This suggests that airborne eDNA methods can serve as a reliable tool for monitoring complex communities where traditional methods may fall short. These methods from that study not only improve the detection rates of bats but also reduce the need for invasive sampling techniques, making it a valuable addition to modern ecological monitoring practices.

### ﻿Anthropogenic threats towards bat studies

Gomantong cave, a dark and humid environment, provides an ideal habitat for millions of swiftlets and bats (Abdullah et al. 2005; Kingston 2010). Its unique geological features make it particularly attractive. Swiftlets and bats contribute significantly to Sabah’s ecosystem, tourism, and economy (Lim et al. 2012). However, the cave is threatened by both natural and human-induced factors (Lundberg and McFarlane 2012). Human activities, such as swiftlet nest harvesting, have had a long-lasting impact on cave ecosystems, including vandalism on cave walls (Wasti et al. 2020), and have led to substantial disturbance of the bats, which may have affected their populations. Harvesting typically occurs in three annual periods: Gomantong cave: February to April, August, and December; meanwhile, Madai cave: April to May, August to September, and November to December. Activities like hacking into the cave walls to install ladders may affect the cave’s integrity (Lundberg and McFarlane 2012). While swiftlet nest harvesting has enriched the local economy, the long-term sustainability of the cave has been overlooked (Idrees and Pradhan 2016). To protect the cave and its resources, a comprehensive understanding of its internal structure is crucial for informed decision-making and management. It would be valuable to establish a long-term monitoring program to track bat populations at these important caves. Historical quantitative data on bat populations from these caves are not available, but anecdotal information suggests large declines in populations of some species. For example, harp traps set along the trails leading to Semud Hitam formerly captured hundreds of individuals per trap in one night (C.M. Francis pers. comm. 31 March 2025), much higher numbers than were observed in this study.

Bats play a vital role in maintaining ecological balance by dispersing seeds, pollinating plants, promoting genetic diversity, and enriching cave ecosystems with nutrients (Sritongchuay and Bumrungsri 2016; Aziz et al. 2017b; Aziz et al. 2021). Additionally, by consuming insect pests, bats help control agricultural pests, reducing the reliance on harmful pesticides (McCracken et al. 2012). Caves are often used for ecotourism, which can educate people about the environment and generate economic benefits (Tanalgo and Hughes 2021). For example, watching flying foxes in Terengganu, Malaysia, has shown that wildlife tourism can raise awareness about bats and promote positive attitudes towards them, which can contribute to their conservation (Roslan et al. 2017). Like other wildlife tourism initiatives, bat-watching can educate people about bats and support sustainable local development, as long as it is done responsibly and with care. However, cave tourism can also pose threats to bat populations. Unsustainable tourism practices, such as excessive noise, light pollution, and disturbance, can have detrimental effects on bat physiology, behavior, and reproductive success (Speakman et al. 1991; Cardiff et al. 2009; Furey and Racey 2016; Lim et al. 2018; Tanalgo et al. 2018).

## ﻿Conclusions

This study has compiled a comprehensive checklist of bat species recorded from selected caves in Sabah and Sarawak. Among the surveyed sites, Madai cave and Gomantong caves were identified as biodiversity hotspots, hosting at least 30 and 26 bat species, respectively. Collectively, surveys around other caves in Borneo found a total of 100 species of bats known in Borneo. The high species richness observed around these caves is likely attributable to extensive sampling efforts, which increased species detectability. The findings underscore the ecological importance of caves as critical habitats for endemic and rare species. Notably, three Bornean endemic species and nine species listed as Near Threatened, six as Vulnerable, and one as Endangered were documented. This highlights the need for targeted conservation measures, especially given the declining population trends of many species and the need for reassessment of the conservation status of several species due to taxonomic updates.

The study also revealed significant biases in species detectability influenced by trapping methods and sampling designs. While harp traps proved effective for capturing small insectivorous species, the integration of acoustic surveys and innovative techniques such as environmental DNA (eDNA) analysis is recommended for future studies. These methods offer the potential to capture a more comprehensive picture of bat diversity while reducing the need for invasive sampling.

Caves such as Gomantong and Madai face significant anthropogenic threats, primarily from intensive nest harvesting activities and prolonged human presence within the caves, as well as habitat degradation due to surrounding forest conversion. Conservation strategies must prioritize sustainable management practices, such as regulating harvesting activities and visitor access, to safeguard these fragile ecosystems. Furthermore, ecotourism initiatives that promote public education about bats can help foster positive attitudes and contribute to long-term conservation goals. By updating the DarkCideS 1.0 database for cave-dwelling bats, this study provides valuable baseline data for future monitoring and conservation prioritization. Future research should aim to integrate machine learning for species identification, expand sampling efforts, and employ advanced monitoring techniques to enhance the understanding of bat diversity and their ecological roles in Borneo.

## ﻿Additional information

### Use of AI

Artificial intelligence tools were employed solely for editorial purposes, specifically to support grammar refinement and language clarity.

### Author ORCIDs

Nur Ain Awatif Mohd-Kanapiah https://orcid.org/0009-0007-0896-6802

Noor Haliza Hasan https://orcid.org/0000-0003-0319-9417

### Data availability

All of the data that support the findings of this study are available in the main text.
